# Purifying selection acts on coding and non-coding sequences of paralogous genes in *Arabidopsis thaliana*

**DOI:** 10.1186/s12864-016-2803-2

**Published:** 2016-06-13

**Authors:** Robert D. Hoffmann, Michael Palmgren

**Affiliations:** Center for Membrane Pumps in Cells and Disease - PUMPKIN, Danish National Research Foundation, Department of Plant and Environmental Sciences, University of Copenhagen, 1871 Frederiksberg C, Denmark

**Keywords:** *Arabidopsis thaliana*, Conserved non-coding sequences, Evolution, Gene expression, mRNA, Paralogous genes, Promoters, 3′ UTR

## Abstract

**Background:**

Whole-genome duplications in the ancestors of many diverse species provided the genetic material for evolutionary novelty. Several models explain the retention of paralogous genes. However, how these models are reflected in the evolution of coding and non-coding sequences of paralogous genes is unknown.

**Results:**

Here, we analyzed the coding and non-coding sequences of paralogous genes in *Arabidopsis thaliana* and compared these sequences with those of orthologous genes in *Arabidopsis lyrata.* Paralogs with lower expression than their duplicate had more nonsynonymous substitutions, were more likely to fractionate, and exhibited less similar expression patterns with their orthologs in the other species. Also, lower-expressed genes had greater tissue specificity. Orthologous conserved non-coding sequences in the promoters, introns, and 3′ untranslated regions were less abundant at lower-expressed genes compared to their higher-expressed paralogs. A gene ontology (GO) term enrichment analysis showed that paralogs with similar expression levels were enriched in GO terms related to ribosomes, whereas paralogs with different expression levels were enriched in terms associated with stress responses.

**Conclusions:**

Loss of conserved non-coding sequences in one gene of a paralogous gene pair correlates with reduced expression levels that are more tissue specific. Together with increased mutation rates in the coding sequences, this suggests that similar forces of purifying selection act on coding and non-coding sequences. We propose that coding and non-coding sequences evolve concurrently following gene duplication.

**Electronic supplementary material:**

The online version of this article (doi:10.1186/s12864-016-2803-2) contains supplementary material, which is available to authorized users.

## Background

A striking difference between metazoans and plants is the recent occurrence of whole genome duplication (WGD) events in plants [1–3]. At least three WGD events have been confirmed in the ancestry of *Arabidopsis thaliana* [4, 5], with the most recent one (entitled alpha; α) occurring around 23 Mya [4]. After a polyploidization event, the genome reorganizes and, although many duplicated sequences are deleted, a considerable proportion of duplicated genes remains as paralogs in the genome [1]. *A. thaliana* contains more than 2500 paralogous gene pairs, accounting for about one-sixth of all protein-coding genes in this species [1, 6]. Due to the wealth of paralogous gene pairs arising from WGD and the reduced selection pressure on redundant gene copies, WGD is thought to provide the potential for adaptive radiation and evolutionary innovations [7–10].

Several models of evolution following a WGD event have been proposed, the most prominent of which are balanced gene drive [11], subfunctionalization of gene pairs [12], and neofunctionalization [9, 13] (reviewed in [14]). The balanced gene drive model is based on the gene balance hypothesis, which predicts that duplicates are retained when the duplication leads to a new balance between the products of dosage-dependent genes [15]. For instance, when the proteins encoded by paralogous genes function as part of a protein complex, the loss of one paralog would change the strength or nature of interactions in the complex, and therefore both copies are likely to be retained [11, 16]. Subfunctionalization describes the process of dividing an ancestral gene function between the two members of a paralogous gene pair. Accordingly, fulfilling the ancestral function now requires duplicate genes [17]. Mutations that lead to new functions of duplicated genes can occur in both protein-coding and non-coding regions [9, 18, 19], and the functional classes of paralogs are suggested to be linked to gene expression [20].

As predicted by the balanced gene drive model, genes encoding subunits of protein complexes or enzymes of the same metabolic pathway tend to be retained after WGD, as shown in ciliates [21, 22], yeast [23], and plants [24]. Genes involved in developmental processes, regulation of transcription, and signal transduction are preferentially retained as duplicates [18, 25–28]. These functional categories suggest that neo-/subfunctionalization drive retention of the duplicates. Stress-responsive genes were found to be retained after WGD, suggesting that environmental challenges promote biased duplicate retention [29]. When paralogs were separated into pairs with similar or differential expression, it was found that DNA- and nucleic acid-binding were overrepresented among similarly expressed paralog pairs, while functions related to biosynthesis and metabolism were overrepresented among the differentially expressed pairs [20]. During the course of evolution, paralogs diverge in amino acid sequence [21] and gene expression profile [18]. Furthermore, paralog coexpression correlates with the number of shared regulatory motifs [30–32]. However, how the coding and non-coding sequences of the same paralog evolve is unknown.

Orthologous conserved non-coding sequences (CNSs) are a characteristic of eukaryotic genomes. As these sequences of non-coding DNA are evolutionarily conserved across species [33, 34], they are thought to have a biological function [35]. Such CNSs are located in introns, intergenic regions proximal or distal to genes, and the 5′ and 3′ untranslated regions (UTRs) of genes. Comparative genomic studies have identified thousands of CNSs in the genomes of humans and model organisms such as mouse and *A. thaliana* [36–41]. In plants, CNSs have been hypothesized to affect the transcription levels of neighboring genes [33, 42] and several studies have shown that CNSs are enriched for transcription factor binding sites [36, 40, 43–45]. Published CNS datasets often overlap to a limited degree only [46], depending on the included species and the detection parameters. Four studies report the identification of CNSs using *A. thaliana* as a reference [36, 38–40]. Baxter et al. (2012) aligned four dicot species and reported 1865 CNSs in the region upstream of the transcription start site (TSS) of 1643 genes [36]. Hupalo and Kern (2013) identified CNSs in 20 angiosperm species using deep whole-genome alignment [39]. Haudry et al. (2013) aligned the genomes of nine members of the Brassicaceae family, identifying over 90,000 CNSs [38]. Van de Velde et al. (2014) used phylogenetic footprinting and whole-genome alignment to identify CNSs in 12 dicot species [40].

In this study, we aimed to identify differences between WGD-derived paralog pairs with similar expression levels and those with different expression levels. We found that the genes of differentially expressed paralog pairs with reduced expression are under less purifying selection, exhibit more tissue-specific expression, and have lost orthologous CNSs compared with paralogs that have equal or increased expression. Paralog pairs with similar expression strength may be retained by gene-dosage constraints, while neo- and/or subfunctionalization may drive retention of differentially expressed pairs.

## Results

### Classification of paralogous genes based on expression levels

**Fig. 1:**
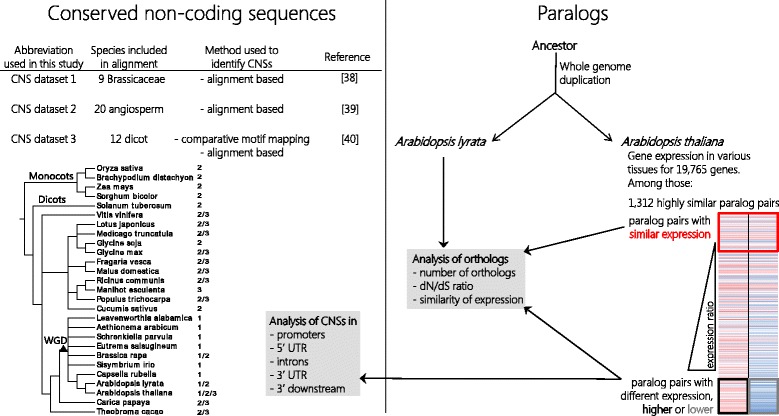
Flowchart of the analyses described in this paper. Based on their relative expression levels (red, higher expression; blue, lower expression), paralogous pairs were grouped into those having similar (red box; log_2_-ratio < 1.25) or differential (log_2_-ratio ≥ 7) expression. Differentially expressed paralogs were further grouped into “higher expression” (black box) or “lower expression” (gray box). Conserved non-coding *A. thaliana* sequences reported in three published studies were analyzed for their presence at paralogous gene pairs

To estimate the average gene transcript levels in *A. thaliana*, we averaged the expression levels of 19,765 genes in 15 tissues or cell types [47] (Additional file 1: Table S1). Among these genes, we identified a set of 1312 highly similar paralogs resulting from the recent α-WGD event in *A. thaliana* (Additional file 1: Table S2). We classified paralogous pairs based on their relative expression levels, and grouped together 245 and 156 pairs with similar (log_2_-ratio < 1.25) and differential (log_2_-ratio ≥ 7) expression levels, respectively. Paralog pairs with differential expression were further categorized into those with higher expression and lower expression (Fig. 1). To compare paralogs that exhibited similar expression with those that had higher and lower expression, one gene of every similarly expressed pair was randomly selected (Additional file 1: Tables S2 and S3), and these randomly selected genes were used in further analyses.

### Differentially expressed paralogs are subject to relaxed purifying selection

To characterize the *A. thaliana* genes in each group (i.e., similar expression, higher expression, and lower expression groups), we compared their expression with that of their orthologs in *Arabidopsis lyrata*. The two species diverged after the α-WGD event and the *A. lyrata* genome has been sequenced [48] and transcript level data are available [49].

**Fig. 2:**
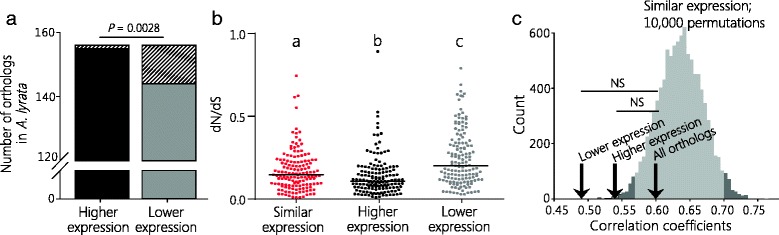
Paralogs with lower expression experience relaxed purifying selection. **a** Paralogs in the higher expression group (black bars) have more orthologs in *A. lyrata* than do their paralogs with lower expression (grey bars) (Fisher’s exact test, two-sided). White hatched bars show number of genes in *A. thaliana* that do not have an ortholog in *A. lyrata*. **b** The dN/dS ratio of *A. thaliana* and *A. lyrata* orthologs was compared between paralogs with similar expression (one paralog of each pair) and paralogs in groups with higher expression and lower expression. Letters indicate significant differences in sample distribution (*P* < 0.001, Kruskal-Wallis test with Dunn’s correction). **c** The gene expression profiles of paralogs with lower expression are less correlated with those of *A. lyrata* orthologs than are paralogs with higher or similar expression. Spearman correlation coefficients based on expression in *A. thaliana* and *A. lyrata* were calculated for paralogs with lower expression (ρ = 0.49, *n* = 109), higher expression (ρ = 0.54, *n* = 147), and all orthologs (ρ = 0.6, *n* = 15,134), indicated by black arrows. The correlation coefficient for differentially-expressed paralogs is different from that for paralogs with similar expression; grey bars show the distribution of ρ values based on 10,000 permutated sets of paralogs from pairs with similar expression (*n* = 245). Light grey bars indicate the distribution of 95 % of the resulting correlation coefficients, and dark grey bars indicate the 2.5 % distribution on either side. For details of the statistical analysis see main text

Firstly, we analyzed how many genes of the higher- and lower-expressed paralogs have orthologs in *A. lyrata*. Amongst the 156 paralogous pairs with differential expression levels, the lower-expressed parologs had 144 orthologs, whereas the higher-expressed paralogs had 155 orthologs (*P* = 0.0028, Fisher’s exact test, two-sided) (Fig. 2a).

Next, to estimate the evolutionary rate of divergence of the paralogous genes’ DNA sequences, we analyzed synonymous (dS) and non-synonymous (dN) substitution rates for *A. thaliana* and *A. lyrata* orthologs. A lower dN/dS ratio is indicative of purifying selection. We detected dN/dS ratios of 0.17, 0.13, and 0.24 for genes in the groups with similar, higher, and lower expression, respectively (*P* < 0.001, Kruskal-Wallis test with Dunn’s correction for multiple testing) (Fig. 2b).

Lastly, we compared gene expression levels using data sampled in flowers at stages 1-14 (*A. lyrata*) and total inflorescences containing flowers at stages 1-14 *(A. thaliana*) [49] (Additional file 1: Table S4). We calculated Spearman correlation coefficients (*ρ*) for all orthologs (*n* = 15,134, *ρ* = 0.6, *P* < 0.0001) and paralogs with higher (*n* = 147, *ρ* = 0.54, *P* < 0.0001) and lower expression (*n* = 109, *ρ* = 0.49, *P* < 0.0001) (Fig. 2c; Additional file 2: Figure S1)*.* Expression correlation between lower- or higher-expressed paralogs and all orthologs was not significantly different (Fig. 2c). We performed the same analysis with ortholog expression data from four species of the Brassicaceae family (*A. thaliana*, *A. lyrata*, *Capsella rubella*, and *Capsella grandiflora*) [50]. The results also showed reduced expression correlation of the lower-expressed paralogs compared to the higher-expressed paralogs (Additional file 2: Figure S2). To further test differences in correlation of gene expression between *A. thaliana* and *A. lyrata* orthologs, we approximated the median correlation co-efficient for the similarly expressed paralogs. As the expression correlation for the similarly expressed paralogs would be different each time we randomly selected one gene from each pair, we computed the correlation coefficients for 10,000 repeated random selections. The resulting correlations had a median *ρ* of 0.64 (with 95 % of values between 0.57 and 0.7), thus overlapping *ρ* of all orthologs, but not those with differential expression (Fig. 2c; Additional file 1: Table S5). Taken together, these findings suggest that the lower-expressed gene of a paralogous pair experiences reduced purifying selection.

### Paralogous genes with different expression levels function in stress responses

We examined the functions of genes in groups with similar and differential expression by performing a gene ontology (GO) term enrichment analysis (Table 1; see Additional file 1: Table S6 for a full list of enriched GO terms). Paralogs with similar average expression levels were found to be enriched for being components of ribosomes, whereas those with differential expression levels were enriched for responses to different abiotic and biotic stresses (Table 1).

**Table 1:** GO term enrichment of paralogous genes with similar or different expression levels

GO term category^c^	Differentially (ratio ≥ 7) expressed^a^			Similarly (ratio < 1.25) expressed^a^		
	FDR^b^	Subset ratio	GO term	FDR^b^	Subset ratio	GO term
Molecular function	0.0230	50 %	catalytic activity			
	0.0360	20 %	hydrolase activity			
	0.0083	14 %	transporter activity			
	0.0100	10 %	substrate-specific transporter activity			
	0.0230	10 %	transmembrane transporter activity			
Biological process	0.0430	48 %	cellular process			
	0.0006	29 %	response to stimulus			
	0.0190	23 %	cellular biosynthetic process			
	0.0330	23 %	biosynthetic process			
	0.0007	19 %	response to stress			
Cellular component	0.0110	36 %	cytoplasm	0.0390	10 %	plasma membrane
	0.0087	35 %	cytoplasmic part	0.0180	7 %	cytosol
	0.0098	26 %	membrane	0.0330	5 %	ribonucleoprotein complex
	0.0017	16 %	plasma membrane	0.0120	4 %	cytosolic part
	0.0110	36 %	cytosol	0.0300	4 %	ribosome

^a^For each pair, the gene with higher expression was selected for analysis

^b^False Discovery Rate (FDR)

^c^For each GO term category, the five entries with the highest Subset ratio are shown

**Fig. 3:**
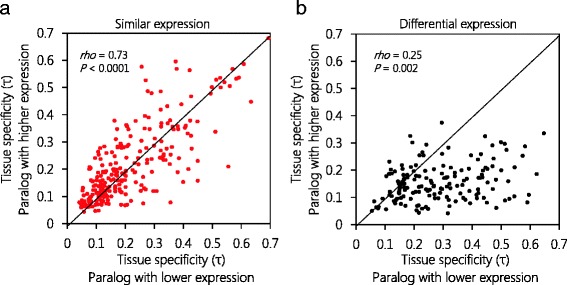
Differentially expressed paralogous pairs diverge in tissue specificity. Paralogous pairs were selected for similar (log_2_-ratio < 1.25, *n* = 245) or differential (log_2_-ratio ≥ 7, *n* = 156) average expression levels. **a** Tissue specificity (τ) is correlated for the two genes of a pair with similar expression levels. **b** Lower-expressed genes of pairs with differential gene expression have stronger tissue specificity than do their higher-expressed paralogs

We reasoned that the stress response is often a rather local action, restricted to certain tissues. Tissue specificity can be measured with the index τ [51], for which values approaching 0 indicate broad gene expression and those approaching 1 indicate tissue-specific expression. We found that expression maxima for 22 % of differentially expressed paralogs were in the same tissue/cell type for both genes (Additional file 1: Table S7). Interestingly, we found that paralogous genes with similar average expression levels had strongly correlating degrees of tissue specificity, whereas the lower-expressed genes of differentially expressed paralogous pairs were skewed towards tissue-specific expression (Fig. 3). This finding suggests that members of differentially expressed paralogous pairs did not simply experience a reduction in average gene expression, but gained tissue-specific expression.

### Number of CNSs in promoter regions, introns, and 3′ UTRs correlates with relative gene expression

**Fig. 4:**
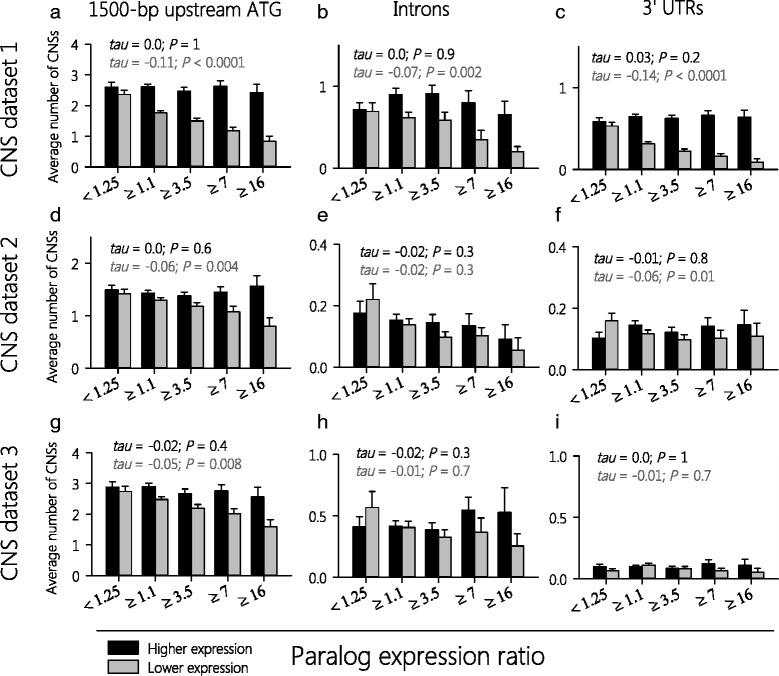
With increasing expression divergence, lower-expressed paralogs have fewer CNSs in their promoters, introns, and 3′ UTRs. The two genes of each paralogous pair were classified as higher-expressed (black bars) or lower-expressed (grey bars) paralog, and plotted according to their ratio in gene expression levels (based on log_2_-values). Y-axes show the average number of CNSs within promoters (1500-bp upstream ATG -100 bp) (**a**, **d**, **g**), introns (**b**, **e**, **h**), and 3′ UTRs (**c**, **f**, **i**). Three different CNS datasets were analyzed [38–40]. A significant negative correlation between the paralog expression ratio and number of CNSs in promoters (Kendall rank correlation) was found for the lower expression group in all datasets. A correlation between paralog expression ratio and number of CNSs in introns or 3′ UTRs was limited (**b**, **c**, **f**). The higher expression group had no significant correlation with the number of CNSs. Error bars are given as s.e.m

Since CNSs are indicative of *cis*-elements that regulate transcription, we compared the number of CNSs associated with paralogous genes in each group. For paralogs with higher expression, no significant correlation was observed between the number of CNSs in the promoter regions and the paralogous pair expression ratio (i.e., the ratio of paralog expression levels; Fig. 4; Additional file 1: Table S8). By contrast, for lower-expressed parolgs, we found a significant negative correlation between the number of CNSs in the promoter regions and diverging paralog expression levels. The correlation was strongest for CNSs of dataset 1 (Kendall’s *tau-b* = 0.11, *P* < 0.0001), but was observed for CNSs obtained from all three CNS datasets (Fig. 4).

Similar results were obtained for CNSs in introns and 3′ untranslated regions (3′ UTRs). In CNS dataset 1, we detected a negative correlation between the paralogous pair expression ratio and the number of CNSs in introns from the lower-expressed gene (Kendall’s *tau-b* = -0.07, *P* = 0.002) but not the number of CNSs from the higher-expressed gene (Kendall’s *tau-*b = 0.0, *P* = 0.9) (Fig. 4; Additional file 1: Table S9). CNSs from datasets 2 and 3 (which are from phylogenetically more distant dicots or angiosperms) showed no significant correlation with gene expression. For paralog expression ratio and CNSs in 3′ UTRs, we found negative correlations between the expression of the lower-expressed gene of a paralogous pair and the number of CNSs in CNS dataset 1 (Kendall’s *tau-b* = -0.14, *P* < 0.0001) and CNS dataset 2 (Kendall’s *tau-b* = -0.06, *P* = 0.01) (Fig. 4). As with CNSs in the promoter regions and introns, higher-expressed paralogs were not significantly correlated with the number of 3′ UTR CNSs.

It is possible that large stretches of conserved sequence in the genome of a common ancestor were fragmented into several shorter CNSs [52, 53], resulting in a miscalculation of the number of CNSs present today. Therefore, we tested the correlation between divergence in gene expression and the sum of bases that form CNSs at any given gene. The results are similar to those obtained in the analysis of single-element CNSs (Additional file 2: Figure S3), suggesting that CNSs likely are not fragments of elements that were previously larger.

In conclusion, we observed that an increase in differential gene expression level is negatively correlated with the number of CNSs located 5′ upstream, in introns, and in 3′ UTRs of the lower-expressed member of the pair.

### CNSs at similarly and differentially expressed genes have comparable properties

**Fig. 5:**
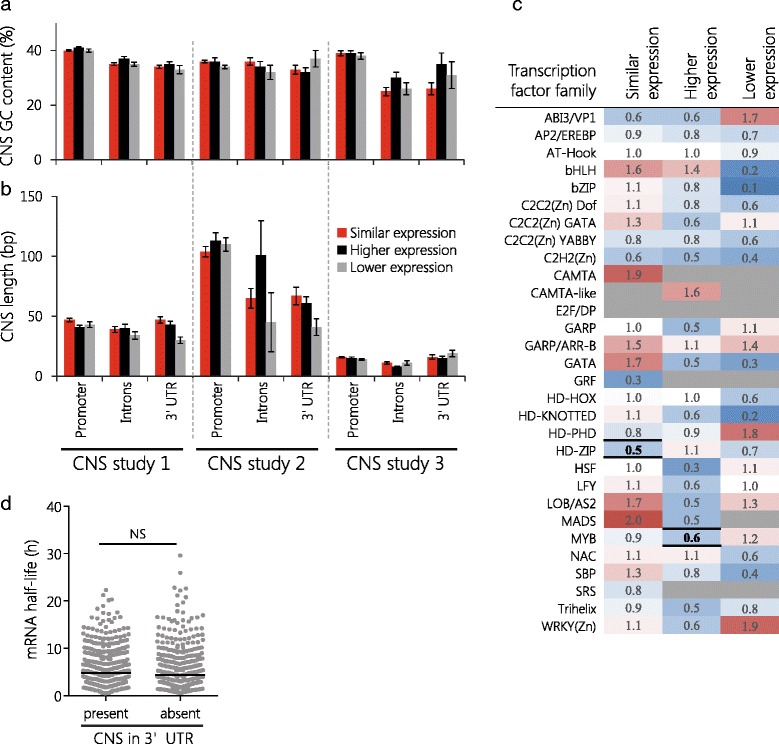
Length, GC content, transcription factor binding sites, and effect on mRNA half-life are similar for CNSs at paralogs. GC content (**a**) and length (**b**) of CNSs from three datasets are compared between paralogs with similar (red), higher (black), or lower (grey) expression in promoters, intronic regions, and 3′ UTRs. No statistically significant differences were found (resampling without replacement, 1000 iterations). Error bars are given as s.e.m. **c** Transcription factor binding site enrichment (red) and depletion (blue) within CNSs located in promoter regions is shown. CNSs are from CNS dataset 1. Missing values (grey) indicate complete absence of the transcription factor binding site motif from CNSs in the respective paralog group. Significant depletion was found for MYB binding site motifs in the higher expression group and HD-ZIP binding site motifs in the similar expression group (resampling without replacement, 1000 iterations; *P* < 0.01). **d** Paralogs with no (absent) or at least one (present) CNS in their 3′ UTRs show no difference in mRNA half-lives (Wilcoxon signed-rank test; black line indicates median value). NS = not significant

We next analyzed if transcription factor (TF) binding motifs were enriched or depleted in CNSs located in promoter regions. For this, we mapped the positions of binding motifs from 274 TFs, belonging to 30 families, within CNSs from CNS dataset 1 (since this dataset is based on the most closely related species). We found that TF binding motifs of several families were enriched or depleted in CNSs present at paralogs in the groups of similar, higher, or differential expression. However, from 90 measurements, only two showed statistically significant deviations (*P* < 0.01; resampling without replacement; 1000 iterations) (Fig. 5c). Paralogs with higher expression are depleted (0.6 fold) of MYB TF binding motifs, and similarly expressed paralogs are depleted (0.5 fold) of HD-ZIP TF binding motifs.

Having identified a negative correlation between paralog expression ratio and 3′ UTR CNSs, we examined whether 3′ UTR CNSs (identified from CNS dataset 1) influence mRNA stability. For this, we compared the mRNA half-life data of paralogous pairs that had one paralog that lacked CNSs in the 3′ UTR with those of paralogous pairs that had one paralog with one or more 3′ UTR CNSs (*n* = 365, Fig. 5d). No significant difference in the corresponding mRNA half-lives was found (Wilcoxon signed-rank test, *P* = 0.15) (Additional file 1: Tables S10 and S11).

We lastly compared the average length and GC content of CNSs from all three CNS datasets in the promoter regions, introns, and 3′ UTRs (Fig. 5a and b). For paralogs in the groups of similar, higher, and lower expression, differences in either CNS length or GC content were small and not statistically significant (*P* < 0.01; resampling without replacement; 1000 iterations).

## Discussion

The aim of this study was to elucidate if and how the retention of paralogs is connected to gene expression. The results provide evidence for a concurrent purifying selection on coding and noncoding sequences of paralogous genes in *A. thaliana*.

### Mutation rates in coding sequences

In our study, we separated two paralogous genes based on their expression strength and measured dN/dS values by comparing each gene with its ortholog in the *A. lyrata* genome. This allowed us to gauge the evolutionary rate of two paralogous genes individually. We found that the lower-expressed genes have acquired more nonsynonymous mutations. This is supported by the finding that paralogs with more similar expression have lower sequence diversity than do paralogs with differential expression [20]. Also, lower-expressed paralogs are more likely to be lost in *A. lyrata*, suggesting that the lost orthologs were under neutral selection. Considering all this, the results suggest that the lower-expressed paralogs are under less purifying selection than are the higher- and similarly expressed paralogs.

### Mutation rates in non-coding sequences

Paralogous genes with similar expression strength were found to have similar tissue specificity. Genes of differentially expressed pairs, however, had diverged in tissue specificity. Higher-expressed genes were broadly expressed, whereas their lower-expressed paralogs were more tissue specific, which is similar to the expression pattern reported for paralogs functioning in stress responses [54]. In the gene expression correlation analysis of *A. thaliana* paralogs and their Brassicaceae orthologs, we found that the expression profiles of lower-expressed paralogs were less correlated with those of their Brassicaceae orthologs than were those of the higher- and similarly expressed paralogs. This suggests conservation in expression profiles, but not for lower-expressed paralogs, possibly encoded by regulatory DNA sequences [30, 32]. That these differences were statistically not significant, though observed in two independent datasets and three species, might be due to a limited availability of expression data for the non-model organisms.

We used orthologous CNSs as a measure of conservation for the non-coding sequences of paralogous genes. This made it possible to analyze the conservation for each gene of a paralogous pair individually. We found that the lower-expressed genes have fewer CNSs in their promoters, introns, and 3′ UTRs, compared to their higher-expressed paralogs. Thus, we assume that the non-coding sequences of the lower-expressed paralogs have higher mutation rates [54]. The ratio of differential expression was negatively correlated with the number of CNSs for genes with lower expression, suggesting that paralog expression divergence is linked to losses of *cis*-regulatory elements residing in CNSs.

### Drivers of paralog retention following the α-WGD event

The retention of duplicated genes following WGD has been explained by several models [14]. Our analysis has revealed that paralogous genes with differential expression are enriched for functions related to responses to different abiotic and biotic stresses. This finding supports the notion that polyploidy is a means to increase adaptability to changing environmental conditions [9], in agreement with the neo- and subfunctionalization models [18]. Indeed, we found that the lower-expressed paralogs of differentially expressed pairs were expressed in a more tissue-specific manner, which has been shown to facilitate neofunctionalization [55].

By contrast, the products of paralogous pairs with highly similar expression levels were enriched for proteins that are subunits of ribosomes. Paralogous genes of *A. thaliana* 80S ribosomal proteins have been shown to be retained by purifying selection and haploinsufficiency [46], indicating that our findings support the notion of gene dosage sensitivity. The balanced gene drive model predicts similar transcript dosage [11, 14]. Accordingly, gene expression profiles must also be similar, and this we found to be the case (Fig. 3).

### CNSs possibly function as promoter and enhancer elements

We showed that CNSs are negatively correlated with increasing expression divergence between two paralogs. This effect was most striking for CNSs identified among nine Brassicaceae species (CNS dataset 1), which was also the most closely related group of organisms analyzed. CNSs in promoter regions are enriched for transcription factor binding sites [33, 38, 44, 45] and hence the correlation we observed between CNSs and paralog transcript level ratios could be attributed to DNA elements promoting transcription. Our analysis of TF binding motif enrichment in CNSs identified only two cases that were statistically significant. One of these was the depletion of MYB binding motifs in higher-expressed paralogs. MYB TFs are regulators of processes ranging from primary and secondary metabolism, over cell fate and identity to developmental processes and responses in biotic and abiotic stresses [56], making it difficult to discern the reason for the depletion we found. Similarly-expressed paralogs were depleted for HD-ZIP binding motifs. HD-ZIP TFs participate in organ development and are involved in responses to environmental conditions [57]. This may explain why HD-ZIP binding motifs are depleted at similarly expressed paralogs, which we found to be enriched for functioning as constituents of ribosomes. Notably, the GC content and length of CNSs were similar for all paralogs, suggesting that the significant depletion of TF binding motifs is not an artefact of general sequence differences.

In introns, CNSs are frequently located in regions flanking exons, suggesting a function in splicing regulation [38], which has been reported to diverge between paralogs in *A. thaliana* [58]. It is more difficult to fathom how the transcript level of a gene is affected by CNSs in the 3′ UTRs. In the datasets available to us, we did not find evidence that mRNA half-lives are influenced by the presence or absence of CNSs in the 3′ UTRs, which has been reported for paralogous CNSs [59]. Alternatively, elements in the 3′ UTRs may act as transcriptional enhancers [60].

## Conclusions

Widely accepted models such as the ‘balanced gene drive’ and ‘neo- and subfunctionalization’ explain the retention of paralogous genes, but it is not known if these models apply to the coding and non-coding sequences of the same genes. Our data link these models of paralog retention to gene function, coding and non-coding sequences, and gene-expression profiles. Because gene expression profiles are in part established by *cis*-regulatory elements inside CNSs, we propose that similar forces of purifying selection act on coding and non-coding sequences. Taken together, our finding that lower-expressed paralogs have fewer CNSs and are more tissue specific than are higher- or similarly expressed paralogs suggests that CNSs promote or enhance transcription in a broad range of organs or cell types in *A. thaliana*. This is different from findings in metazoans, where CNSs regulate transcription in specific cells [61, 62].

## Methods

### Sequence data and definitions of gene-associated regions

All data for the *A. thaliana* genome sequence were downloaded from TAIR [63, 64]. Promoter regions were defined as 1500 bp, 1000 bp, and 500 bp upstream of start codons, but omitting ATG -100 bp. 5′ UTRs were defined as the regions 100 bp upstream of the start codon (to rule out any bias for highly expressed genes with well-annotated 5′ UTRs) [65]. Introns encompass all introns of the representative gene model that lie within the protein coding region. 3′ UTRs were defined as the regions 200 bp downstream of stop codons [61]. 3′ downstream regions were defined as the regions 300 to 1000 bp downstream of stop codons.

### Gene expression data and measurement of tissue specificity

Robust Multi-array Average (RMA)-normalized microarray data for 79 diverse samples of *Arabidopsis thaliana* tissues and cell types harvested at different developmental stages [47] were downloaded from ArrayExpress [66]. The data were disregarded for probes that detect genes other than those annotated as ‘protein-coding’ (TAIR10) and probes that hybridize with more than one gene, and also for cases where several probes were annotated as hybridizing to the same gene (according to _at to AGI Conversion Tool [67]). Expression values (obtained in *A. thaliana* accession Col-0) for samples of the same organ were then averaged, namely roots (all samples), green parts of seedlings (7- and 8-days-old), leaves (all samples except data for ‘senescing leaves’ but including ‘cauline leaf’), seeds (all samples, stages 6-10), siliques (all samples, stages 3,4, and 5, with seeds), stem (1^st^ node and 2^nd^ internode), and flowers (all samples, stages 9, 10/11, 12, 15, and one undefined). Data for pedicels, sepals, petals, stamen, carpels (all from flowers at stage 15), hypocotyls, cotyledons, and mature pollen were kept as individual samples (Additional file 1: Table S1). To approximate the overall average gene expression level, the average across the fifteen tissue- or cell type-specific samples was calculated. The tissue specificity for every gene was calculated with the index τ [51]:

$$ \tau =\frac{{\displaystyle {\sum}_{i=1}^N}\left[1-{x}_i\right]}{N-1} $$

where *N* is the number of tissues and *x*_*i*_ is the expression profile component normalized by the maximal component value. Genes with a τ of close to 0 are broadly expressed across all tissues, while those with tissue-specific expression approach τ = 1.

### Paralogous gene pairs

A list of 2563 paralogous gene pairs that evolved after a whole genome duplication (WGD) event 23 Mya was downloaded from a previous publication [6]. Gene pairs that lacked an annotation in TAIR10 for one or both of the genes were excluded. To enable cross-platform analysis of the gene pairs, only those present in the gene expression data (see above) were selected. To strengthen the gene pairs’ similarity with respect to the proteins they encode, all pairs with more than 5 % difference in protein coding sequence length (relative to the longer protein; TAIR10) were filtered out. These adjustments resulted in a set of 1312 paralogous pairs that was used for analysis in this study (Additional file 1: Table S4). Paralogous pairs were grouped as similar or differentially expressed when the expression ratio between the two genes of a pair was < 1.25 (*n* = 245) or ≥ 7 (*n* = 156), respectively. For further analysis of the similarly expressed paralogs, one gene per pair was randomly selected, resulting in a list of paralogous genes with “similar expression” (Additional file 1: Table S3). Differentially expressed pairs were divided into sets of “higher expression” or “lower expression”.

### Substitution rates and orthologs

The BioMart [68] tool in EnsemblPlants [69] was used to retrieve the dN (non-synonymous; change in protein sequence) and dS (synonymous; protein sequence unchanged) data from genes in *A. lyrata* that are orthologs of the genes in *A. thaliana* described above. Ensembl uses codeml from PAML (Phylogenetic Analysis by Maximum Likelihood) [70] to calculate dN and dS values. The obtained data were cleared for non-representative *A. thaliana* gene models. Where one *A. thaliana* gene had more than one ortholog in *A. lyrata*, only the ortholog with higher ‘*A. lyrata* % identity’ values was retained. If these values were identical, the entry with the lowest dS value was used. Statistical analysis using the Kruskal-Wallis test and Dunn’s correction for multiple comparisons was carried out in Prism 6 (GraphPad Software, Inc.). The list of orthologs identified for the *A. thaliana* paralogs was also used to quantify the differentially expressed *A. thaliana* paralogs that had an *A. lyrata* ortholog.

### A. lyrata *gene expression data*

Gene expression data for *A. thaliana* and *A. lyrata* orthologs in the form of *Z* scores were provided by the authors [49]. Briefly, for *A. thaliana*, RNA was extracted from whole inflorescences, up to stage 14 flowers, and applied to a tiling array. Triplicate biological samples were generated, with a single technical replicate per sample. For *A. thaliana*, mRNA was extracted from floral tissues (stages 1-14), followed by sequencing (mRNAseq). Two technical replicates each of two biological replicates were sequenced. To make comparisons between the two gene expression datasets, which were identified using different methods, the distribution of expression values was standardized. Data were log-normalized and the resulting normal distribution of gene expression levels was transformed into standard (*Z*) scores, i.e., units of SD from the mean [49].

Statistical analysis (Spearman rank correlation, two-sided) of the correlation between *Z* score expression data for all genes in our analysis and the sets of paralogs with higher expression and lower expression was performed with the function cor.test in R [71, 72]. To compare the Spearman correlation coefficients, *ρ* was calculated for all orthologs, *ρ* values were treated as though they were Pearson correlation coefficients (*r*), and Fisher’s *z*-transformation was used to determine significant differences between two correlation coefficients [73] using VassarStats [74]. Differences in similarly expressed paralogous pairs were further analyzed if *Z* scores were obtainable for both genes. To approximate the correlation between *A. thaliana* and *A. lyrata* gene expression for the similarly expressed genes, an R script (Additional file 2: Script 1) was written that randomly selected expression values from one gene of each paralogous pair and calculated the Spearman correlation coefficient. This was repeated 10,000 times.

### GO annotation analysis

Genes from the higher expression and similar expression datasets were analyzed for GO term enrichment using AgriGO [75, 76]. Statistical analysis for GO term enrichment of gene sets was performed using Fisher’s exact test with subsequent adjustment for multiple testing by calculating the false discovery rate (FDR) [77]. The minimum number of mapping entries was set to 5, and the list of 19,765 genes derived from the ATH1 microarray was used as a reference.

### Conserved non-coding sequence data

Published data for conserved non-coding sequences were downloaded from publicly available websites [78–80]. CNS dataset 1: *A. thaliana* CNSs track was selected and CNSs termed sncCNS were filtered out [38, 78]; CNS dataset 2: mostCons track [39, 79]; CNS dataset 3: BED file of all CNSs [40, 80];.All data files were imported to Google BigQuery [81] and CNSs were retrieved within the 500 bp, 1000 bp, and 1500 bp region upstream of the start codon (ATG -1), in any intron, 200 bp downstream of the stop codon, and within 300 to 1000 bp downstream of the stop codon. Where a neighboring gene extended into the specified upstream or downstream regions (examined using TAIR10 data for intergenic sequences), the queried sequence was shortened accordingly. Only CNSs that were situated entirely within the respective regions were considered for further analyses. If a particular sequence did not contain any CNS, this was counted as zero. Statistical analyses of the correlation (Kendall rank correlation, *tau-b,* which adjusts for tied values) between the number of CNSs (or the sum of bases of all these CNSs) and transcript levels (as log_2_-values), or fold-change between two paralogous genes, were performed using the function cor.test in R [71, 72].

### Analyses of CNSs

The average length and GC content of CNSs from each CNS dataset and each genetic region (promoter, intronic, or 3′ UTR; as defined above) were tested for significance by resampling 1000 times without replacement; the sample pools were promoter regions of all 19,765 genes in the analysis. Data for transcription factor (TF) binding sites of 274 TFs, belonging to 30 TF families, were obtained from AthaMap [82, 83] and uploaded to Google BigQuery [81]. For further analysis, only those TF binding sites were considered that had a sequence conservation score of at least 50 %. TF binding sites were counted as being inside a CNS (in 1500-bp promoter regions) if the site position started no more than 2 bases upstream of a CNS, or had at least 4 bases overlapping with a CNS. Significance was tested by resampling 1000 times without replacement; the sample pool was defined as CNSs in promoters of all 19,765 genes in the analysis. Resampling was performed with the Resampling Stats for Excel add-in (statistics.com, LLC).

### mRNA half-life data

Data of mRNA half-lives sampled in *A. thaliana* suspension cell cultures were downloaded from the supplemental material accompanying the publication [84]. Paralogous pairs were further analyzed only when half-life data were available for the mRNAs encoded by both genes. Pairs were further selected if one gene lacked a CNS in its 3′ UTR and the other had at least one CNS in its 3′ UTR. Statistical analysis (Wilcoxon signed-rank test) was performed in Prism 6 (GraphPad Software, Inc.).

## Additional files

Additional file 1: Table S1. Expression values and TAU (tissue specificity) values of *A. thaliana* genes. Table S2. Paralogous gene pairs in *A. thaliana*; their expression levels and TAU values. Table S3. List of one randomly selected gene from every similarly expressed paralogous pair. Table S4. Normalized expression values of *A. thaliana* and *A. lyrata* orthologs. Table S5. rho values from 10,000 permutations. Table S6. Full list of significantly enriched GO terms. Table S7. Tissue with highest gene expression for differentially expressed paralogous genes. Table S8. CNSs found at *A. thaliana* genes. Table S9. Statistics for the correlation between the number of CNSs at paralogous genes and expression levels. Table S10. mRNA half-life data for genes included in this study. Table S11. mRNA half-life data for paralogous pairs that have 3′ UTR CNSs in one gene only. (XLSX 6431 kb) Additional file 2: Figure S1. Correlation between gene expression levels of orthologs in *A. thaliana* and *A. lyrata*. Correlation between gene expression in the lower expression (a) and higher expression (b) data sets measured in *A. thaliana* and *A. lyrata*. Figure S2. Correlation between gene expression levels of orthologs in four Brassicaceae species. Correlation between gene expression in the higher expression (a-c) and lower expression (d-f) data sets measured in *A. thaliana* and *A. lyrata* (a, d), *A. thaliana* and *C. rubella* (b, e), and *A. thaliana* and *C. grandiflora* (c, f). Figure S3. Correlation between differential gene expression and the number of conserved nucleotides in the promoters, introns, and 3′ UTRs of paralogous pairs. The number of conserved nucleotides in CNSs within the 1500-bp upstream regions (a, d, g), introns (b, e, h), or 3′ UTRs (c, f, i) of all genes present on the ATH1 microarray was determined using three different CNS datasets. For every gene, the average transcription level (log_2_-value) is given on the y-axis. For lower-expressed paralogs, CNSs and transcription levels are negatively correlated (a, b, c, f, and g). Script 1: R script to calculate rho-values. (PDF 490 kb)
