# Supplementary material for: Purifying selection acts on coding and non-coding sequences of paralogous genes in Arabidopsis thaliana
Source: BMC Genomics. 2016 Jun 13;17:456. doi: 10.1186/s12864-016-2803-2 (PMC4906602; doi:10.1186/s12864-016-2803-2)
Supplement: Additional file 2: Figure S1. — Correlation between gene expression levels of orthologs in A. thaliana and A. lyrata. Correlation between gene expression in the lower expression (a) and higher expression (b) data sets measured in A. thaliana and A. lyrata. Figure S2. Correlation between gene expression levels of orthologs in four Brassicaceae species. Correlation between gene expression in the higher expression (a-c) and lower expression (d-f) data sets measured in A. thaliana and A. lyrata (a, d), A. thaliana and C. rubella (b, e), and A. thaliana and C. grandiflora (c, f). Figure S3. Correlation between differential gene expression and the number of conserved nucleotides in the promoters, introns, and 3′ UTRs of paralogous pairs. The number of conserved nucleotides in CNSs within the 1500-bp upstream regions (a, d, g), introns (b, e, h), or 3′ UTRs (c, f, i) of all genes present on the ATH1 microarray was determined using three different CNS datasets. For every gene, the average transcription level (log2-value) is given on the y-axis. For lower-expressed paralogs, CNSs and transcription levels are negatively correlated (a, b, c, f, and g). Script 1: R script to calculate rho-values. (PDF 490 kb) [file 12864_2016_2803_MOESM2_ESM.pdf]

Additional file 2

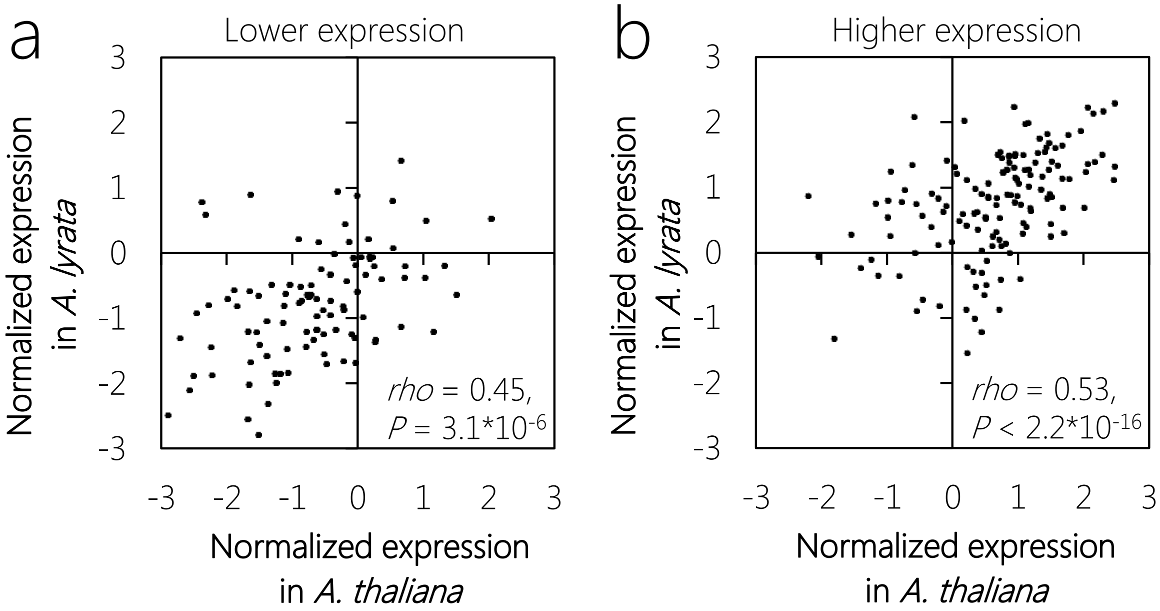

Figure S1: Correlation between gene expression levels of orthologs in *A. thaliana* and *A. lyrata*.

Correlation between gene expression in the lower expression (a) and higher expression (b) data sets measured in *A. thaliana* and *A. lyrata*.

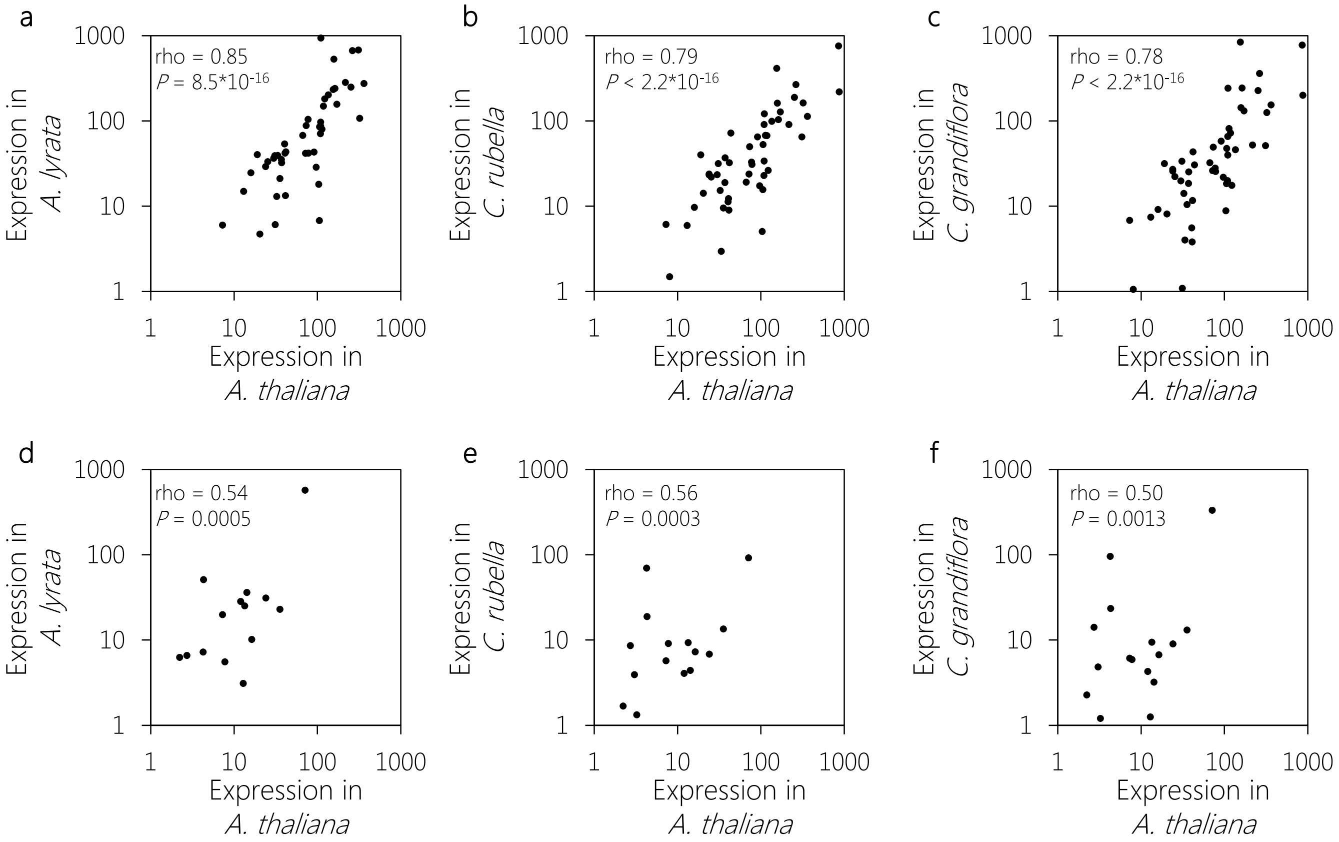

Figure S2: Correlation between gene expression levels of orthologs in four Brassicaceae species.

Correlation between gene expression in the higher expression (a-c) and lower expression (d-f) data sets measured in *A. thaliana* and *A. lyrata* (a, d), *A. thaliana* and *C. rubella* (b, e), and *A. thaliana* and *C. grandiflora* (c, f).

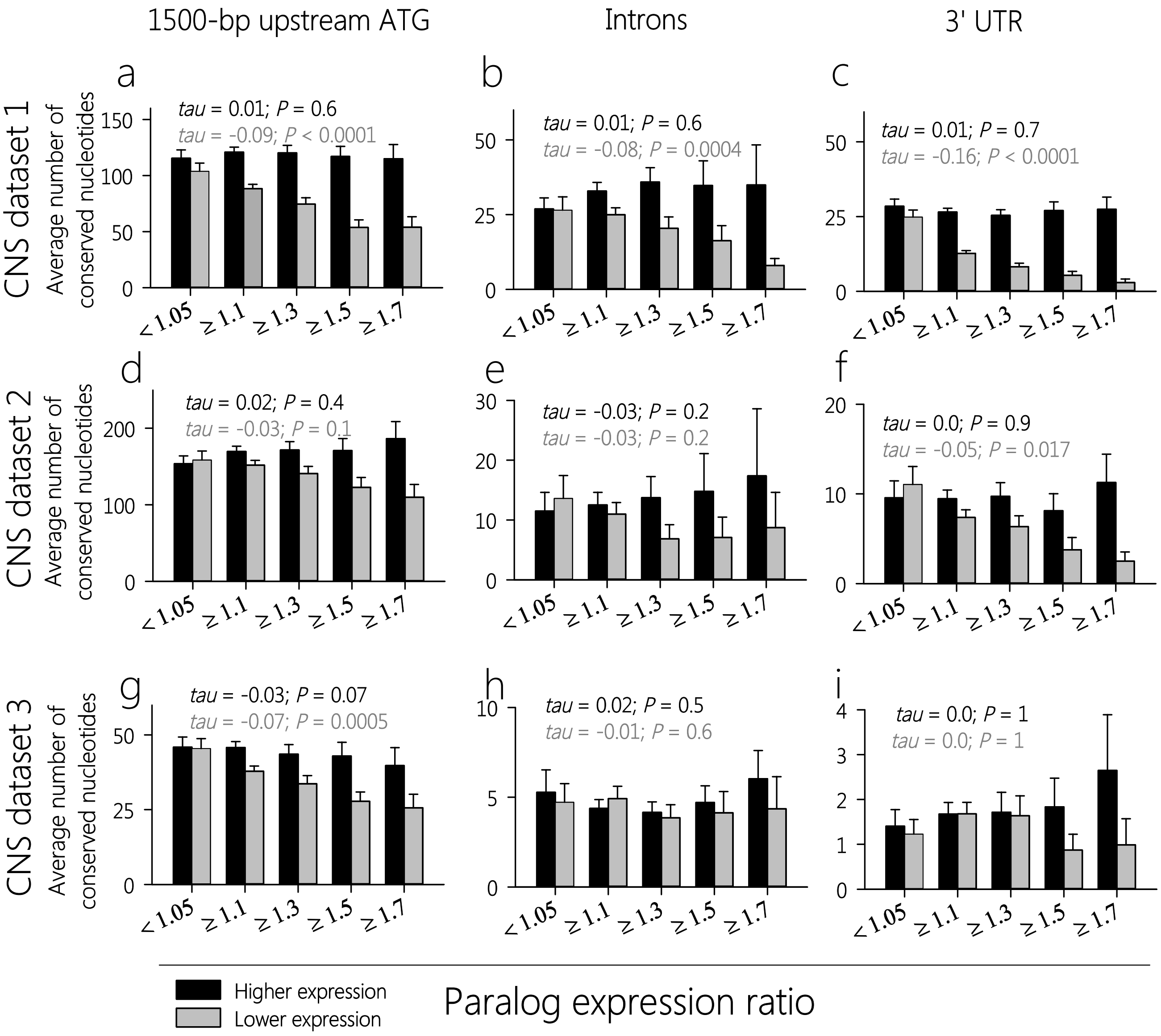

Figure S3: Correlation between differential gene expression and the number of conserved nucleotides in the promoters, introns, and 3' UTRs of paralogous pairs.

The number of conserved nucleotides in CNSs within the 1,500-bp upstream regions (a, d, g), introns (b, e, h), or 3' UTRs (c, f, i) of all genes present on the ATH1 microarray was determined using three different CNS datasets. For every gene, the average transcription level (log_2-value) is given on the y-axis. For lower-expressed paralogs, CNSs and transcription levels are negatively correlated (a, b, c, f, and g).

Script 1: R script to calculate rho-values.

```
#clean memory#
rm(list=ls())

#read the dataset#
#data in the first four columns are organized as A_x, A_y, B_x, B_y#
data<-read.table("C:\\example.csv",sep=";",head=T)

#generate an empty vector of length 10000 to fill with rho parameter estimates#
rho_estimates<-rep(0,10000)

#generate 10000 loops#
for (j in 1:length(rho_estimates)){

#generate a sequence of number with values randomly assigned between 1 and 2 (only integers)#
#if the first value is 1 I'll fill the first row of the dataset with A_x and A_y, if it's 2 B_x and B_y and so on#
number_seq<-sample(1:2,length(data[,1]),replace=T)

#generate an empty dataset of 2 variables which I'll fill with either A_x and A_y or B_x and B_y
X_Y=data.frame(X=rep(0,length(number_seq)),Y=rep(0,length(number_seq)))

# this loop says if the i_th value of number_seq is 1 take the first 2 columns of the starting dataset,
otherwise take the others#
for (i in 1:length(number_seq)){

if (number_seq[i]==1)  X_Y[i,]<-data[i,1:2] else X_Y[i,]<-data[i,3:4]


}
#calculate test and fill the J-th position of the vector of estimates#
rho_value<-cor.test(X_Y[,1],X_Y[,2],method="spearman")
rho_estimates[j]<-rho_value$estimate

}
```
